# Machine Learning-Enhanced Structure-Based Gaussian Expansion for Efficient Wavepacket Calculations

**DOI:** 10.1021/acs.jpclett.5c01254

**Published:** 2025-06-07

**Authors:** Takumi Koshiba, Manabu Kanno, Fuminori Misaizu, Hirohiko Kono

**Affiliations:** Department of Chemistry, Graduate School of Science, 13101 Tohoku University, Sendai 980-8578, Japan

## Abstract

The theoretical treatment of molecular wavepackets remains computationally demanding and becomes increasingly impractical for complex systems with a large number of atoms. To tackle this problem, we previously developed the structure-based Gaussian (SBG) expansion method, where space-fixed Gaussian basis functions for the expansion of wavepackets are placed intensively around reaction pathways connecting equilibrium structures and transition states. In this study, we incorporated two machine learning techniques into the SBG expansion, thereby developing a highly efficient and versatile approach for wavepacket calculations: the principal component analysis for systematic construction of the SBG basis set and the Gaussian process regression for interpolation of potential energy surfaces. To demonstrate the performance of this approach, we constructed full-dimensional nuclear wave functions for the umbrella inversion tunneling in H_3_O^+^. The improved expansion using 33 SBG bases successfully reproduced the experimental vibrational energies up to overtone excited states with only 19 quantum chemical calculations. We also confirmed the feasibility for larger systems through the applications to intramolecular hydrogen transfer in 9-hydroxyphenalenone and its asymmetrically deuterated species.

## Graphical abstract

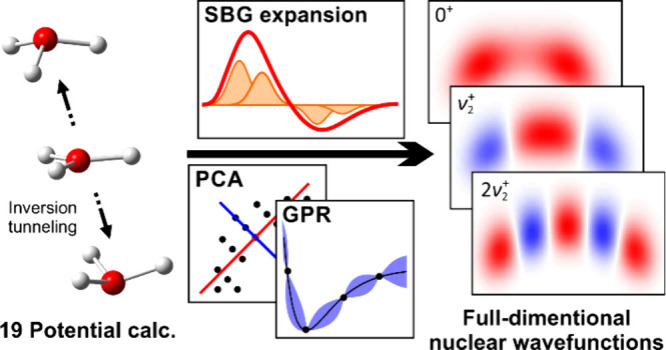

Nuclear wavepackets are essential for achieving a comprehensive understanding of the quantum (static and dynamic) nature of nuclear motion in molecules including molecular vibrations and chemical reactions. Among various methods developed for calculating nuclear wavepackets, the multiconfiguration time-dependent Hartree (MCTDH) method^1^ is the most successful approach. In the MCTDH, nuclear wavepackets are expressed as a linear combination of Hartree products of so-called single-particle functions represented on a grid for a small subset of the system’s degrees of freedom. To date, several updated versions of the MCTDH, such as the multilayer^2^ and on-the-fly^3^ formulations, have been reported. However, even with the MCTDH, the exponential increase of computational cost cannot be completely avoided, and calculating nuclear wavepackets remains computationally demanding.

In contrast to grid-based methods requiring global potential energy surfaces (PESs), Gaussian-type basis functions allow us to evaluate the system Hamiltonian using only local potential information around the bases. Moving (time-dependent)^4,5^ or space-fixed (time-independent)^6–9^ Gaussians can be used to expand nuclear wavepackets; we adopt the latter for higher numerical stability.^10,11^ We have developed the structure-based Gaussian (SBG) expansion method,^11^ in which space-fixed Gaussian bases are placed intensively in important regions for describing target reactions such as equilibrium structures, transition states, and reaction pathways. Additionally, the width parameters of the bases are determined individually from the local shape of PESs at the structures. These features of the SBG expansion enable efficient calculations of full-dimensional nuclear wavepackets in molecular systems. The SBG expansion has been applied to intramolecular hydrogen transfer through tunneling in malonaldehyde and has successfully reproduced the experimental values of the ground-state tunnel splitting with relatively lower computational costs.

In this Letter, we introduce two machine learning techniques to the SBG expansion method for more efficient nuclear wavepacket calculations. First, the principal component analysis (PCA) is employed for the automatic selection of coordinates with large contribution to the reaction. The selected coordinates are utilized for the efficient construction of the basis set for expanding nuclear wavepackets. Second, PESs are interpolated using the Gaussian process regression (GPR) technique to reduce the number of quantum chemical (QC) calculations. The improved method is applied to calculate the vibrational eigenstates of H_3_O^+^ (the hydronium ion), 9-hydroxyphenalenone (9HP-*d*_0_), and its asymmetrically deuterated species (9HP-*d*_1_). We demonstrate that these techniques significantly accelerate nuclear wavepacket calculations while maintaining accuracy. The applicability of the SBG expansion is thereby extended to large molecules, making wavepacket calculations more feasible for complex systems.

In the SBG expansion, a nuclear wavepacket χ­(***Q***, *t*) is expanded in terms of *L* space-fixed Gaussian bases {*G*_*i*_(***Q***)}:

1 $$\begin{array}{c} \chi \left(Q,t\right)=\overset{L}{\underset{i=1}{\sum }}c_{i}\left(t\right)G_{i}\left(Q\right) \end{array}$$

Here, ***Q*** = {*Q*_1_, *Q*_2_, *Q*_3_, ..., *Q*_3*N*_}^T^ is the vector of mass-weighted Cartesian coordinates of an *N*-atom system, *t* is time, and *c*_*i*_(*t*) are expansion coefficients. The 3*N*-dimensional Gaussian bases are represented as

2 $$\begin{array}{c} G_{i}\left(Q\right)=\exp\left[-{\left(Q-Q_{i}\right)}^{T}\alpha_{i}\left(Q-Q_{i}\right)+\zeta_{i}\right] \end{array}$$

where ***Q***_*i*_ is the central position, **α**_*i*_ is the width parameter, and ζ_*i*_ is the normalization constant of the *i*th basis. In the present study, by analogy to the wave functions of a 3*N*-dimensional harmonic oscillator, we determine **α**_*i*_ from the 3*N* × 3*N* local Hessian matrix ***V*″** at ***Q***_*i*_:

3 $$\begin{array}{c} \alpha_{i}=\frac{1}{2\hbar }\sqrt{V^{\prime \prime }\left(Q_{i}\right)} \end{array}$$

**α**_*i*_ determined by eq 3 can achieve higher accuracy than the one proposed in our previous study^11^ by incorporating interatomic coupling motions. ***V*″**(***Q***_*i*_) has six zero-eigenvalues corresponding to the translation and rotation, making Gaussian integrals incomputable. Moreover, at nonequilibrium structures, it may include negative eigenvalues corresponding to imaginary-frequency vibrational modes, which preclude the definition of $\sqrt{V^{\prime \prime }\left(Q_{i}\right)}$ . To avoid these problems, we convert ***V*″**(***Q***_*i*_) into a positive-definite matrix by the following operations: the eigenvalues close to zero are replaced with a finite positive constant *V*_const_^″^, and the signs of the negative eigenvalues are flipped to accurately capture the steep shape of χ­(***Q***,*t*) in the potential region with a negative curvature. To obtain reasonable results, *V*_const_^″^ must be set to an appropriate value. Detailed discussions are presented in .

The total kinetic energy operator *T̂*_total_ of the molecule can be written using the corresponding momentum operator ***P̂***_total_:

4 $$\begin{array}{c} {\mathrm{T̂}}_{\mathrm{total}}=\frac{1}{2}{\mathrm{P̂}}_{\mathrm{total}}^{T}{\mathrm{P̂}}_{\mathrm{total}}=-\frac{\hbar^{2}}{2}\overset{3N}{\underset{j=1}{\sum }}\frac{\partial^{2}}{\partial^{2}Q_{j}} \end{array}$$

We focus on the vibrational motion, excluding the contributions from translational and rotational motions in *T̂*_total_. In our previous study,^11^ the translational contributions were removed by defining the translational kinetic energy operator *T̂*_tra_, and the rotational ones were partly removed by aligning molecules so that no angular momenta were generated for shifts from structure to structure. In addition to these operations, in the present study, we introduce the rotational kinetic energy operator *T̂*_rot_ to remove the rotational contributions adequately. The mathematical expressions of *T̂*_tra_ and *T̂*_rot_ are

5 $$\begin{array}{c} {\mathrm{T̂}}_{\mathrm{tra}}=\frac{1}{2}{\mathrm{P̂}}_{\mathrm{total}}^{T}{Ô}_{\mathrm{tra}}^{T}{Ô}_{\mathrm{tra}}{\mathrm{P̂}}_{\mathrm{total}}=\frac{1}{2}{\mathrm{P̂}}_{\mathrm{total}}^{T}{Ô}_{\mathrm{tra}}{\mathrm{P̂}}_{\mathrm{total}} \end{array}$$

6 $$\begin{array}{c} {\mathrm{T̂}}_{\mathrm{rot}}=\frac{1}{2}{\mathrm{P̂}}_{\mathrm{total}}^{T}{Ô}_{\mathrm{rot}}^{T}{Ô}_{\mathrm{rot}}{\mathrm{P̂}}_{\mathrm{total}}=\frac{1}{2}{\mathrm{P̂}}_{\mathrm{total}}^{T}{Ô}_{\mathrm{rot}}{\mathrm{P̂}}_{\mathrm{total}} \end{array}$$

Here, ***Ô***_tra_ and ***Ô***_rot_ are the translational and rotational projection operators, respectively. For the explicit forms of ***Ô***_tra_ and ***Ô***_rot_, see Section IV in ref 12. The vibrational kinetic energy operator *T̂*_vib_ is then given by

7 $$\begin{array}{c} {\mathrm{T̂}}_{\mathrm{vib}}={\mathrm{T̂}}_{\mathrm{total}}-{\mathrm{T̂}}_{\mathrm{tra}}-{\mathrm{T̂}}_{\mathrm{rot}} \end{array}$$

Potential energy matrix elements are evaluated under the local harmonic approximation:

8 ⟨Gi|V̂|Gi′⟩≈⟨Gi|V(Qi,i′)+12(Q̂−Qi,i′)TV″(Qi,i′)(Q̂−Qi,i′)|Gi′⟩={V(Qi,i′)+14Tr[(αi+αi′)−1V″(Qi,i′)]}⟨Gi|Gi′⟩

*V*(***Q***_*i*,*i′*_) is the potential energy at ***Q***_*i*, *i′*_ defined as the peak position of the product of *G*_*i*_ and *G*_*i′*_:

9 $$\begin{array}{c} Q_{i,i^{\prime }}={\left(\alpha_{i}+\alpha_{i^{\prime }}\right)}^{-1}\left(\alpha_{i}Q_{i}+\alpha_{i^{\prime }}Q_{i^{\prime }}\right) \end{array}$$

In this Letter, to demonstrate the performance of the present method, we apply it to the calculation of the vibrational eigenstates of H_3_O^+^ and 9HP. The time-independent Schrödinger equation can be written in the matrix form

10 $$\begin{array}{c} S^{-1}H_{\mathrm{vib}}c_{m}=S^{-1}\left(T_{\mathrm{vib}}+V\right)c_{m}=E_{m}c_{m} \end{array}$$

Here, ***T***_vib_ and ***V*** are the matrices of the corresponding operators, and ***S*** is the overlap matrix. The elements of these matrices can be calculated analytically. Those of ***T***_rot_ are provided in the and the others are in the SI of ref 11. *E*_*m*_ and ***c***_*m*_ are the *m*th vibrational eigenenergy and eigenvector, respectively, obtained by diagonalizing ***S*** ^–1^ ***H***_vib_.

In cases where basis functions are employed to expand nuclear wavepackets, the arrangement of the bases strongly influences computational results. The straightforward yet effective choice is to place bases along the intrinsic reaction coordinate (IRC). The IRC is the steepest descent path from a transition state to equilibrium structures, providing fundamental insights into chemical reactions. In many cases, wavepackets predominantly propagate along the IRC as the reaction progresses. Thus, we begin by placing bases along the IRC. However, this basis set alone is insufficient for accurate calculations because pathways away from the IRC also contribute to the reaction, for example, via tunneling or dynamical effects. To account for these contributions, additional bases must be placed around the IRC.

To prepare a set of additional bases, we introduce auxiliary vibrational coordinates. One of the most fundamental candidates is local normal modes, which give 3*N* – 7 vibrational coordinates orthogonal to the IRC. In our previous study,^11^ additional bases were distributed along all local normal modes around the basis centers ***Q***_*i*_ on the IRC using a scheme similar to that described below. The number of additional bases around each ***Q***_*i*_ was reduced to approximately the square of the number of vibrational degrees of freedom. However, as the molecular size grows, the number of local normal modes increases, and consequently, that of additional bases rises significantly, leading to a huge computational cost. A promising solution is to select only essential coordinates. In the case of hydrogen tunneling in malonaldehyde,^11^ we made this selection based on our chemical intuition. Intuitive selections, however, are impractical for reactions where dominant motions are not obvious and may overlook essential contributions. To overcome this problem, we adopt PCA in this study to automatically identify coordinates that significantly contribute to the reaction.

PCA is a linear transformation method that determines axes maximizing structural variances, commonly used for dimensionality reduction by selecting high-variance variables. In the field of physical chemistry, it has been applied to analyze and visualize reaction pathways and trajectories obtained by molecular dynamics simulations.^13^ PCA diagonalizes the covariance matrix ***C*** derived from the structural dataset {***Q***_*l*_^′^} along the IRC:

11 $$\begin{array}{c} \Lambda =U^{T}\mathrm{CU} \end{array}$$

12 $$\begin{array}{c} C=\underset{l}{\sum }\left(Q_{l}^{\prime }-{\mathrm{Q̅}}^{\prime }\right){\left(Q_{l}^{\prime }-{\mathrm{Q̅}}^{\prime }\right)}^{T} \end{array}$$

Here, ***Q̅*** **′** is the averaged structure of {***Q***_*l*_^′^}, where the total number of *l* is much larger than that of the bases on the IRC. ***U*** is the orthogonal matrix composed of the eigenvectors of ***C***, which are called principal components. The *n*th principal component is abbreviated hereafter as PC*n*. The eigenvalue λ_*n*_ = **(Λ)**_*n, n*_ corresponds to the variance along PC*n*. When λ_*n*_ is large, significant structural changes are induced along PC*n* during the reaction, and thus we need to add bases along PC*n*. In contrast, for small λ_*n*′_, the deformation along PC*n*′ in the reaction is negligible and can be adequately described by the bases on the IRC. Note that the variances λ_*n*_ and the eigenvectors PC*n* are common across all basis centers ***Q***_*i*_ on the IRC, while local normal modes are defined at each of them.

Using PCA, we follow the steps below to generate additional bases:

(1) Calculate λ_*n*_ and PC*n* to select essential coordinates with large λ_*n*_.
(2) Determine distances ***d***_*i*,*n*_ along PC*n* from each ***Q***_*i*_ on the IRC so that the overlaps of the existing basis at ***Q***_*i*_ with the bases added at ***Q***_*i*_ ± ***d***_*i*,*n*_ (first-order shifts) become appropriate (0.7–0.8 in most cases) for all selected *n*. In the overlap calculations at this step, the width parameters of the additional first-order shifted bases are tentatively assumed to be the same as that of the existing basis at ***Q***_*i*_, i.e., **α**_*i*_.
(3) Place further additional bases at the points shifted from ***Q***_*i*_ by ±(***d***_*i*,*n*_ ± ***d***_*i*,*n′*_) (second-order shifts).
(4) Recalculate the width parameter of each additional basis using ***V*** **″** at its central position. Then, compute the overlap matrix again with the updated width parameters and remove the bases having large overlaps with those on the IRC or other additional bases.

We can systematically verify the accuracy of this approach by lowering the threshold value of λ_*n*_ for selection and checking the convergence of the calculated results.

In the local harmonic approximation shown in eq 8, the evaluation of potential energy matrix elements requires potential energies *V*(***Q***_*i*,*i′*_) and Hessian matrices ***V*** **″**(***Q***_*i*,*i*′_). This potential information must be calculated not only at the central points {***Q***_*i*_} of each basis (including additional ones) but also at the middle points {***Q***_*i*,*i*′_} of each basis pair. The SBG expansion with *L* bases requires up to *L*(*L* + 1)/2 QC calculations, making it a significant computational bottleneck.

To effectively reduce the number of QC calculations, we here propose employing a machine learning technique for the interpolation of PESs. Potential information is obtained by QC calculations only at the *L* central points and estimated by machine learning at the *L*(*L* – 1)/2 middle points. Since the machine learning estimation is used exclusively as interpolation, which is inherently more reliable and practical than extrapolation,^14^ high computational accuracy can be expected with an appropriate choice of machine learning techniques.

In this study, we adopt GPR among various machine learning techniques available for potential interpolation. GPR is a type of kernel regression method and has been applied to the construction of PESs.^3,15,16^ While GPR is a relatively simple method, it is highly versatile and resistant to overfitting, a common issue that many machine learning techniques often face. In addition, there has been a report^17^ that it can achieve accuracy comparable to more complex methods such as neural networks.

We train the GPR model by the potential information ***Y*** = {*Y*_*i*_} at the central positions {***Q***_*i*_} of bases; *Y*_*i*_ is *V*(***Q***_*i*_) or an element of ***V*** **″**(***Q***_*i*_), i.e., ∂^2^ *V*/∂*Q*_*j*_∂*Q*_*j*′_|_***Q***_*i*__. The potential information Y_*_ at the middle points Q_*_ of basis pairs is obtained by the following simple matrix calculation:

13 $$\begin{array}{c} Y_{*}=K{\left(\left\{Q_{i}\right\},Q_{*}\right)}^{T}{\left[K\left(\left\{Q_{i}\right\},\left\{Q_{i}\right\}\right)+\sigma^{2}1\right]}^{-1}Y \end{array}$$

14 $$\begin{array}{c} K\left(\left\{Q_{i}\right\},Q_{*}\right)=\left(\begin{array}{c} k\left(Q_{1},Q_{*}\right)\\ k\left(Q_{2},Q_{*}\right)\\ \vdots \end{array}\right) \end{array}$$

15 $$\begin{array}{c} K\left(\left\{Q_{i}\right\},\left\{Q_{i}\right\}\right)=\left(\begin{array}{ccc} k\left(Q_{1},Q_{1}\right) & k\left(Q_{1},Q_{2}\right) & \cdots \\ k\left(Q_{2},Q_{1}\right) & k\left(Q_{2},Q_{2}\right) & \cdots \\ \vdots & \vdots & \ddots \end{array}\right) \end{array}$$

Here, σ is the regularization constant, **1** is the identity matrix, and *k*(***Q***_*i*_, ***Q***_*i*′_) is a kernel function representing the correlation between two data. In this study, we use a simple Gaussian kernel function

16 $$\begin{array}{c} k\left(Q_{i},Q_{i^{\prime }}\right)=\tau^{2}\;\exp\left(-\frac{{||Q_{i}-Q_{i^{\prime }}||}^{2}}{2\beta^{2}}\right) \end{array}$$

Hyperparameters (τ, β) in the kernel function are optimized by a grid search based on the leave-one-out cross-validation method^18^ to minimize the estimation error in the sum of the potential energies and Hessian terms in eq 8.

To start with, we applied this method to the evaluation of tunnel splitting in H_3_O^+^ as a benchmark. Given that tunneling in H_3_O^+^ has been extensively studied both experimentally^19–21^ and theoretically,^22^ our results can be directly compared with the existing literature data. It has a triangular pyramidal structure with two equivalent potential minima along the umbrella inversion. Superpositions of two localized states in these minima form new states with tunnel splitting. High-resolution spectroscopic measurements^19–21^ have determined the values of the tunnel splitting even for overtone excited vibrational states with very complicated wave functions. We aim to reproduce the vibrational energies of these states with the present method, which is capable of constructing full-dimensional wavepackets of the system at low computational costs.

The IRC search was performed at the MP2/aug-cc-pVTZ level of theory, and then potential energies and Hessian matrices were refined at the CCSD­(T)/aug-cc-pVTZ level using the GAUSSIAN16 package.^23^ We used the *V*_const_^″^ value corresponding to a frequency of 700 cm^–1^, which is smaller (but not too much) than the harmonic frequency for the mode of interest (i.e., umbrella inversion). We placed one basis at each of the two minima and three bases along the IRC connecting the minima (five bases equally spaced on the IRC). The overlaps of the adjacent bases were about 0.7, which is in an appropriate range. The number of QC calculations required for the SBG expansion with *L* = 5 is, in principle, *L*(*L* + 1)/2 = 15; in practice, it is reduced to 9 by applying the reflection symmetry of the umbrella inversion. We first calculated the vibrational eigenenergies of H_3_O^+^ using this basis set. The results are listed in the second column (named “IRC”) of Table 1, together with previous experimental^19–21^ and computational^22^ data in the seventh and eighth columns, respectively. All the calculated vibrational energies have large errors from the literature values. The root-mean-square error (RMSE) is as large as 504.59 cm^–1^. As expected, placing bases only on the IRC is insufficient to achieve accurate results for tunneling in H_3_O^+^.

**Table 1:** Calculated Vibrational Energies, RMSE from the Experimental Values, and Zero-Point Energy (ZPE) for H_3_O^+^ (in Units of cm^–1^)­^a^

	IRC	PC1	PC1, 2	PC1, 2/GPR	All vib.	Exp.^19–21^	RVIB4 ^22^
No. of bases	5	11	33	33	329	–	–
No. of QC calc.^a^	9	36	295	19	28337	–	–
ZPE	7554.46	7522.65	7477.13	7474.95	7442.52	–	7451
0^–^	63.43	49.43	45.79	46.95	47.52	55.35	41
ν_2_^+^	651.79	577.89	596.17	594.88	593.83	581.17	580
ν_2_^–^	1304.45	1011.20	989.89	994.08	989.20	954.40	917
2ν_2_^+^	2419.70	1814.47	1496.98	1514.36	1492.20	1475.84	1421
RMSE	504.59	171.72	22.49	28.79	20.62	–	34

a The labels ν_2_ and 2ν_2_ represent the fundamental and overtone, respectively, of the umbrella inversion mode, and 0 denotes the vibrational ground state. The “±” superscripts denote the parity of a pair of tunneling states.

b The number of QC calculations is reduced from *L*(*L*+1)/2 (or *L* in the case of PC1, 2/GPR) by applying the reflection symmetry of the umbrella inversion.

As another reference calculation, we next added bases at the first- and second-order shifted points along all local normal modes. In this case, the total numbers of bases and QC calculations were 329 and 28337, respectively. The calculated results presented in the sixth column (“All vib.”) of Table 1 reproduced the experimental ones very well. Compared with the previous version of the SBG expansion,^11^ the two improvements defined as described in eq 3 and eq 7, the width parameter and the removal of the rotational contributions, significantly enhanced the computational accuracy (see ). The accuracy of the results is comparable with that of the literature values obtained by RVIB4, a theory specialized for describing the vibrational states of tetraatomic molecules, with the CCSD­(T)/aug-cc-pVTZ potential.^22^

Here, to reduce the number of bases from 329, we selected essential coordinates for the inversion in H_3_O^+^ by performing PCA on the structures along the IRC. The variances λ_*n*_ and characters of the resultant PC*n* are summarized in Table 2. Among the 12 degrees of freedom in H_3_O^+^, only two of them have a relatively large λ_*n*_ value. The most dominant coordinate, PC1, corresponds to the umbrella mode as expected. The second dominant coordinate, PC2, represents the symmetric stretching of OH bonds associated with bond elongation in the transition state. This is consistent with a two-dimensional model developed by Miani et al., which suggested that the umbrella and OH symmetric stretching vibrations are sufficient to accurately describe the inversion motion.^24^ The other vibrations such as the asymmetric stretching of OH bonds and the bending motion of HOH angles do not contribute to the IRC since they break the *C*_3_ symmetry of H_3_O^+^. PC3–12 are mixtures of these vibrations with translational and rotational motions. The PCA successfully extracted essential coordinates for tunneling in H_3_O^+^ without any chemical intuition.

**Table 2:** PCA Results of the IRC for the Inversion in H_3_O^+^

	λ_*n*_/λ_1_	character
PC1	1	umbrella inversion
PC2	1.3 × 10^–2^	OH symmetric stretching
PC3–12	<10^–9^	OH asymmetric stretching, HOH bending, rotation, translation

The vibrational energies computed for the cases where bases are added along PC1 and along both PC1 and PC2 are shown in the third and fourth columns (“PC1” and “PC1, 2”) of Table 1, respectively. Adding bases along PC1 resulted in significant improvements for accurately describing the 0^–^ and ν_2_^±^ states. Since PC1 is almost parallel to the IRC, the additional bases are placed in the extended regions of the IRC (see ) to describe the spread of the wave functions in this direction. However, the calculation results indicate that the 2ν_2_^+^ state still exhibits a substantial and persistent error. Such overtone excited states have a complicated wave function, which is very difficult to expand with a small number of bases. When additional bases are placed along both PC1 and PC2, the accuracy is remarkably enhanced, achieving results comparable to those obtained by adding bases along all local normal modes. Notably, this approach reduces the number of bases to just 33, approximately one-tenth of the total required when using all local normal modes. Moreover, the number of QC calculations is about 1/100 of that in the latter case.

In a similar manner to the local normal mode approach, PC*n* can be orthogonalized to the IRC at the center of each basis on the IRC. This transformation hardly affects the computational accuracy for H_3_O^+^ (see ).

To further reduce computational costs, we also conducted potential energy estimation using GPR. The results of the GPR estimation for the case with additional bases along PC1 and PC2 are shown in Figure 1. In Figure 1b, each element in the Hessian matrix ***V*** **″** was estimated to evaluate $\frac{1}{4}\mathrm{Tr}\left[{\left(\alpha_{i}+\alpha_{i^{\prime }}\right)}^{-1}V^{\prime \prime }\left(Q_{i,i^{\prime }}\right)\right]$ in eq 8. The values obtained by QC calculations and the GPR estimation are close to each other, indicating the high accuracy of the GPR estimation. The RMSEs in the potential energies and Hessian terms from the QC calculated values are 12.02 cm^–1^ and 2.59 cm^–1^, respectively.

**Figure 1:**
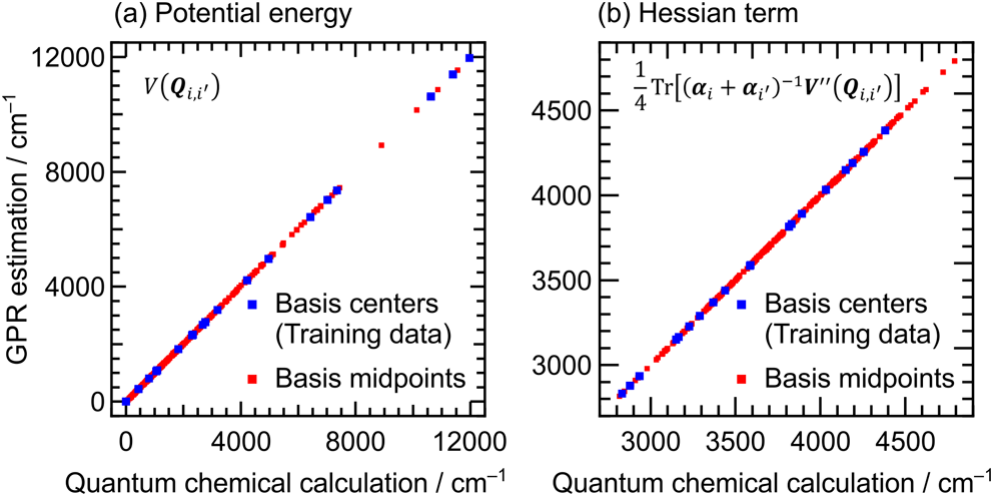
(a) Potential energies and (b) Hessian terms obtained by QC calculations and the GPR estimation at the central positions of bases (blue squares), which are used for training data, and at the middle points of basis pairs (red squares).

We calculated vibrational energies using the interpolated potential information. The results are shown in the fifth column of Table 1 as “PC1, 2/GPR”. They are close to those obtained by calculating all potential information (the fourth column of Table 1) and agree well with the experimental values.

By introducing the GPR estimation, QC calculations are required only at the *L* central points of bases (*L* = 33 in this case). Ultimately, molecular symmetry further reduces the total number of QC calculations required for constructing the full-dimensional wavepackets of H_3_O^+^ to just 19. Compared to the study by Miani et al., which required around 100 QC calculations for a limited two-dimensional model,^24^ our approach achieves comparable results with substantially fewer calculations, underscoring its computational efficiency.

The GPR estimation is applicable to any basis set for the SBG expansion. The results of the GPR estimation for the case with additional bases along all local normal modes are presented in . This basis set contains much more bases (*L* = 329) than the one used in Figure 1 (*L* = 33), and the resultant GPR estimation error is slightly larger (see ). As in this example, the error can become significant in some cases with a large number of bases. This issue arises from the simplicity of the GPR method. We employed GPR as an initial approach but other potential interpolation methods such as neural networks are also promising. These more sophisticated models will further improve both estimation accuracy and numerical stability, particularly when dealing with a large number of bases.

So far, we have focused on the umbrella inversion and therefore derived principal components from the IRC to prepare the optimal basis set for this mode. A great advantage of the SBG expansion is that one can adopt arbitrary appropriate paths and auxiliary coordinates for basis set construction, depending on the problem of interest. For instance, vibrationally excited states and tunnel splittings of the other modes in H_3_O^+^ can be adequately described by distributing bases in the corresponding directions (see the results for the “All vib.” case in ).

Finally, to test the feasibility of the present method for systems with far more than 10 atoms, we applied it to intramolecular hydrogen transfer in 9HP, consisting of 23 atoms (see Figure 2a). The asymmetric deuteration in 9HP-*d*_1_ with respect to hydrogen transfer paths leads to asymmetric wave functions, unlike symmetric 9HP-*d*_0_. In general, the magnitude of secondary isotope effects, arising from isotopic substitutions at sites not directly involved in bond formation or breaking, depends strongly on the molecular framework and the substitution site; it is not obvious when such effects become pronounced in deuterated compounds. To our knowledge, the ground-state tunnel splitting values for isolated 9HP-*d*_0_ and 9HP-*d*_1_ are unknown, while those for 9HP-*d*_0_ in neon^25^ and *n*-hexane^26^ matrices have been observed at 69 cm^–1^ and 85 cm^–1^, respectively. Therefore, we focus not on the accuracy but on the convergence of the calculated results with an increasing number of PC*n* considered. The density functional theory, which is less accurate but much faster than CCSD­(T), was thus adopted at the ωB97X-D/6–31G­(d,p) level in QC calculations using GAUSSIAN 16.

**Figure 2:**
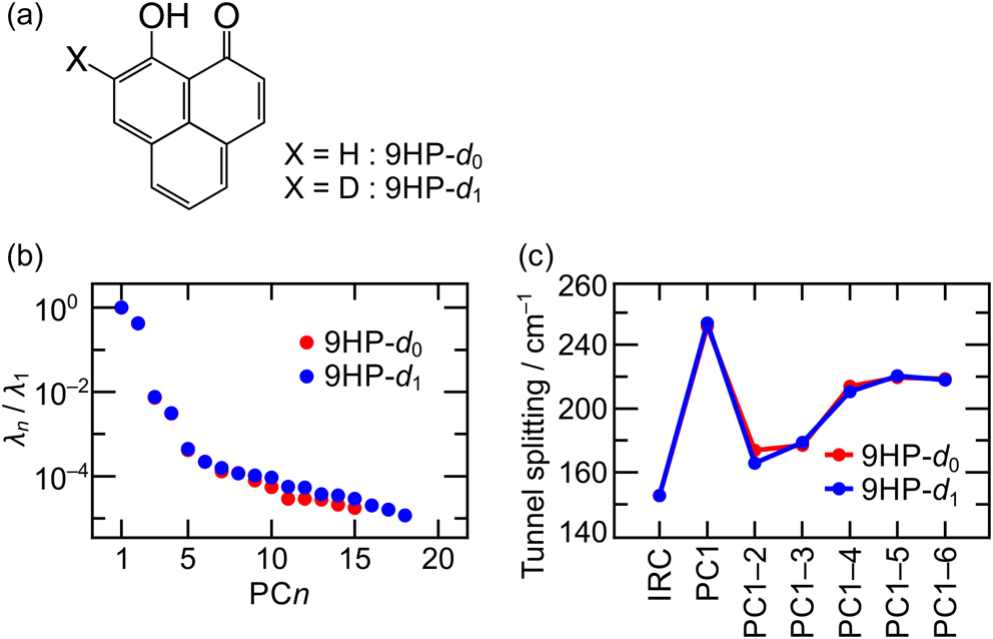
(a) Structural formulas, (b) variances λ_*n*_ along PC*n* of the hydrogen transfer IRC, and (c) ground-state tunnel splitting of 9HP-*d*_0_ an*d* 9HP-*d*_1_.

The values of λ_*n*_ obtained by PCA on the relevant IRC are plotted in Figure 2b. As in the case of H_3_O^+^, λ_*n*_ exhibits an exponential decay with increasing *n*. We have studied some other systems, and the rapid decay is a common trend. This suggests that a limited number of PC*n* are sufficient, even for such large molecules. The *V*_const_^″^ value used corresponds to 2800 cm^–1^, which is slightly smaller than the harmonic frequency for OH stretching related to hydrogen transfer between oxygen atoms. We placed 11 bases on the IRC and added bases sequentially along PC1 to PC6. The GPR estimation was utilized for potential energies and Hessians. The calculated tunnel splittings are displayed in Figure 2c. As expected, by considering only several PC*n*, the splitting converges approximately to 220 cm^–1^ for both 9HP-*d*_0_ and 9HP-*d*_1_. This indicates the possibility that secondary isotope effects on the ground-state tunnel splitting are not significant in 9HP, unlike in malonaldehyde, for which the SBG expansion method predicted noticeable secondary effects of deuterium substitution.^11^ In addition to PC1 and PC2 (hydrogen tunneling motions), PC3 and PC4 (skeletal deformations) are necessary to reach a convergence. The results in Figure 2 illustrate that PCA serves as a powerful tool to automatically extract such nontrivial essential coordinates, especially for large systems. The total number of bases in the “PC1–4” basis set is 289. It is much smaller than 9 × 10^4^ for the case with additional first- and second-order shifted bases along all 63 vibrational degrees of freedom.

In summary, we have developed an efficient and versatile method for nuclear wavepacket calculations by incorporating two machine learning techniques into the SBG expansion method: PCA for automatic extraction of essential coordinates and GPR potential interpolation for reduction in the number of QC calculations. The validity of the improved SBG expansion has been demonstrated through its applications to the evaluation of the vibrational eigenstates of H_3_O^+^ and 9HP. The present method requires only 19 QC calculations to describe full-dimensional nuclear wave functions of H_3_O^+^ even for overtone excited states with complex patterns. A small number of essential coordinates (including nontrivial ones) can also be identified automatically in large and asymmetric systems like 9HP-*d*_0_ and 9HP-*d*_1_, thus enabling efficient calculations of many-dimensional wavepackets. The machine learning-enhanced SBG expansion is expected to be useful for challenging systems such as those with strongly anharmonic PESs; PCA and GPR suppress an increase in computational cost due to the use of multiple narrow Gaussians necessitated by the anharmonicity. To further explore the potential of this method, we have already applied it to a more complex reaction in biological molecules, i.e., the intermolecular double proton transfer reaction in a DNA base pair consisting of about 30 atoms, and successfully captured the proton transfer dynamics with a reasonable computational cost. Details of this application will be reported elsewhere.

## Supplementary Material





## Notes

Section S1, Choice of *V*_const_^″^ for suppressing vibration–rotation couplings; Section S2, Rotational kinetic energy matrix elements; Section S3, Comparison with the previous version of the SBG expansion; Section S4, Orthogonalization of principal components to the IRC; Section S5, Potential interpolation by GPR with a large number of bases; Section S6, Vibrationally excited states for the modes other than the umbrella inversion (PDF)
Transparent Peer Review report available (PDF)
