# Correction to “Machine Learning-Enhanced Structure-Based Gaussian Expansion for Efficient Wavepacket Calculations”

**DOI:** 10.1021/acs.jpclett.5c01841

**Published:** 2025-06-30

**Authors:** Takumi Koshiba, Manabu Kanno, Fuminori Misaizu, Hirohiko Kono

**Affiliations:** Department of Chemistry, Graduate School of Science, 13101 Tohoku University, Sendai 980-8578, Japan

A typo has been identified in the literature data shown in Table 1 of the original Letter.^1^ The zero-point energy of H_3_O^+^ from the RVIB4 calculation by Bowman’s group was indicated as 7451 cm^–1^, but the correct value is 7435 cm^–1^.^2^ This correction does not change any of our calculated results or the conclusions in the Letter.

As a closely related work, it should also be noted that Bowman’s group demonstrated a potential energy surface of H_3_O^+^ based on higher-level ab initio energies mostly at the CCSD­(T)-F12/aug-cc-pVQZ level.^3^ This surface yielded vibrational energies of H_3_O^+^ in better agreement with experimental values.
