# Supplementary material for: Machine Learning-Enhanced Structure-Based Gaussian Expansion for Efficient Wavepacket Calculations
Source: J Phys Chem Lett. 2025 Jun 7;16(24):5986–92. doi: 10.1021/acs.jpclett.5c01254 (PMC12183755; doi:10.1021/acs.jpclett.5c01254)
Supplement: Supplementary file 1 [file jz5c01254_si_001.pdf]

Supporting Information

Machine Learning-Enhanced Structure-Based Gaussian Expansion for Efficient Wavepacket Calculations

Takumi Koshiba, Manabu Kanno,[*,a)] *Fuminori Misaizu, and Hirohiko Kono*[*,b)]

Department of Chemistry, Graduate School of Science, Tohoku University, Sendai 980-8578, Japan.

Corresponding Authors

a) e-mail: manabu.kanno.d2@tohoku.ac.jp

b) e-mail: hirohiko.kono.d6@tohoku.ac.jp

S1. Choice of V''_{const} for suppressing vibration–rotation couplings

As stated in the main text, the value of the parameter V''_{const}, which is introduced to replace the near-zero eigenvalues of the local Hessian matrix $\boldsymbol{V}''$, affects computational results. The zero-point energy (ZPE) of H_3O^+ calculated using two Gaussian basis functions $\{G_i(\boldsymbol{Q})\}$, one at the equilibrium structure $\boldsymbol{Q}_{\mathrm{eq}}$ and the other at the first-order shifted point $\boldsymbol{Q}_{\mathrm{shift}}$ along the asymmetric OH stretching vibration (ν_3), is shown in **Table S1**, together with the value obtained under the harmonic approximation. Since the ν_3 mode breaks the C_3 symmetry of H_3O^+, the shifts along ν_3 strongly induce vibration–rotation couplings. First, with $\sqrt{V''_{\mathrm{const}}} = 10\ \mathrm{cm}^{-1}$, the width parameters $\boldsymbol{\alpha}_i$ of the two bases and the distance between them are determined following Steps 2 and 4 of the basis set construction schemes presented in the main text. The resultant ZPE is much smaller than in the harmonic case, indicating an unphysical behavior. Next, with the distance value unchanged, $\boldsymbol{\alpha}_i$ are recalculated with $\sqrt{V''_{\mathrm{const}}} = 700\ \mathrm{cm}^{-1}$. The ZPE in this case is almost the same as the harmonic one. These results demonstrate the importance of selecting an appropriate V''_{const} value to achieve accurate calculations. In this section, we investigate the reason for the unphysical result with a small value of V''_{const}.

Table S1. ZPE in units of cm^{-1} obtained with two different V''_{const} values in comparison with the harmonic approximation. Quantum chemical (QC) calculations were performed at the CCSD(T)/aug-cc-pVTZ level of theory.

	$\sqrt{V''_{\mathrm{const}}}$ =10 cm^{-1}	$\sqrt{V''_{\mathrm{const}}}$ =700 cm^{-1}	Harmonic
ZPE	5476.38	7629.47	7629.47

Let us project the peak position of the product of G_{eq} and G_{shift}, $\boldsymbol{Q}_{\mathrm{eq,shift}}$, onto the normal modes in H_3O^+ defined at $\boldsymbol{Q}_{\mathrm{eq}}$. The rotational components depend markedly on the V''_{const} value, whereas the translational and vibrational ones are almost unchanged regardless of V''_{const}. For instance, the rotational component (around an axis perpendicular to the C_3 axis) with the largest dependence on V''_{const} and the ν_3 vibrational component evaluated in the above two cases ($\sqrt{V''_{\mathrm{const}}}$ = 10 cm^{-1} and 700 cm^{-1}) are listed in **Table S2**. The difference in $\boldsymbol{Q}_{\mathrm{eq,shift}}$ along the rotation, which depends on V''_{const}, should affect the results of calculations. Hereafter, we focus exclusively on this rotational coordinate and ν_3 to discuss how $\boldsymbol{Q}_{\mathrm{eq,shift}}$ depends on V''_{const}.

Table S2. The projections of $\boldsymbol{Q}_{\mathrm{eq,shift}}$ onto the rotation around an axis perpendicular to the C_3 axis and ν_3 (in atomic units) obtained with two different V''_{const} values.

	$\sqrt{V''_{\mathrm{const}}}$ =10 cm^{-1}	$\sqrt{V''_{\mathrm{const}}}$ =700 cm^{-1}
Rotation	−7.68	0.54
ν_3	4.10	4.15

We transform the mass-weighted coordinates $\boldsymbol{Q}$ into the dimensionless vibrational coordinates $\boldsymbol{Q}'$ as

$$\boldsymbol{Q}' = \boldsymbol{U}^{\mathrm{T}} \boldsymbol{\alpha}_{\mathrm{eq}}^{1/2} \left(\boldsymbol{Q} - \boldsymbol{Q}_{\mathrm{eq}} \right). \tag{S1}$$

Here, $\boldsymbol{U}$ represents the orthogonal matrix diagonalizing $\boldsymbol{\alpha}_{\mathrm{eq}}$. The width parameters $\boldsymbol{\alpha}_i$ are then transformed as

$$\boldsymbol{\alpha}_i' = \boldsymbol{U}^{\mathrm{T}} \boldsymbol{\alpha}_{\mathrm{eq}}^{-1/2} \boldsymbol{\alpha}_i \boldsymbol{\alpha}_{\mathrm{eq}}^{-1/2} \boldsymbol{U}. \tag{S2}$$

One can readily find that $\boldsymbol{\alpha}_{\mathrm{eq}}'$ is the identity matrix, $\mathbf{1}$. Assuming that the difference between $\boldsymbol{V}''\left(\boldsymbol{Q}_{\mathrm{eq}}\right)$ and $\boldsymbol{V}''(\boldsymbol{Q}_{\mathrm{shift}})$ (i.e., between $\boldsymbol{\alpha}_{\mathrm{eq}}'$ and $\boldsymbol{\alpha}_{\mathrm{shift}}'$) is small, $\boldsymbol{\alpha}_{\mathrm{shift}}'$ can be written using small perturbation terms c, c', and c'':

$$\boldsymbol{\alpha}_{\mathrm{shift}}' = \begin{pmatrix} 1 + c' & c \\ c & 1 + c'' \end{pmatrix}. \tag{S3}$$

Since the rotational component of $\boldsymbol{Q}_{\mathrm{shift}}'$ is small (but nonzero, which is introduced to remove angular momenta generated by the shift from $\boldsymbol{Q}_{\mathrm{eq}}'$), $\boldsymbol{Q}_{\mathrm{eq,shift}}'$ is then written as

$$\boldsymbol{Q}_{\mathrm{eq,shift}}' = \left(\boldsymbol{\alpha}_{\mathrm{eq}}' + \boldsymbol{\alpha}_{\mathrm{shift}}' \right)^{-1} \left(\boldsymbol{\alpha}_{\mathrm{eq}}' \boldsymbol{Q}_{\mathrm{eq}}' + \boldsymbol{\alpha}_{\mathrm{shift}}' \boldsymbol{Q}_{\mathrm{shift}}' \right) \approx \begin{pmatrix} \frac{c}{4} Q_{\mathrm{shift}_{\nu 3}}' \\ \frac{1}{2} Q_{\mathrm{shift}_{\nu 3}}' \end{pmatrix}. \tag{S4}$$

Despite the small rotational displacement in $\boldsymbol{Q}_{\mathrm{shift}}'$, that in $\boldsymbol{Q}_{\mathrm{eq,shift}}'$ can be large depending on the coupling term c. Displacements along the rotational coordinates hardly change the molecular structure and thus have little influence on the potential energy $V\left(\boldsymbol{Q}_{\mathrm{eq,shift}}\right)$. However, they can

significantly affect the Hessian term $\frac{1}{4}\mathrm{Tr}\left[\left(\boldsymbol{\alpha}_{\mathrm{eq}}+\boldsymbol{\alpha}_{\mathrm{shift}}\right)^{-1}\boldsymbol{V}''\left(\boldsymbol{Q}_{\mathrm{eq,shift}}\right)\right]$ by changing the directions of the vibrational and rotational eigenvectors of $\boldsymbol{V}''\left(\boldsymbol{Q}_{\mathrm{eq,shift}}\right)$.

The coupling term c is given as

$$c=\frac{\left(\boldsymbol{U}^{\mathrm{T}}\boldsymbol{\alpha}_{\mathrm{shift}}\boldsymbol{U}\right)_{\mathrm{rot},\nu3}}{\sqrt{\sqrt{V''_{\mathrm{const}}}\,\omega_{\nu3}}},\tag{S5}$$

where $\omega_{\nu3}$ is the frequency of ν_3 at $\boldsymbol{Q}_{\mathrm{eq}}$. The origin of c is the change in the width parameter at $\boldsymbol{Q}_{\mathrm{shift}}$ from that at $\boldsymbol{Q}_{\mathrm{eq}}$, $\left(\boldsymbol{U}^{\mathrm{T}}\boldsymbol{\alpha}_{\mathrm{shift}}\boldsymbol{U}\right)_{\mathrm{rot},\nu3}$, and c is reduced by $(V''_{\mathrm{const}})^{-1/4}$. An appropriately large value of V''_{const} suppresses the vibration–rotation coupling and results in accurate vibrational energies.

S2. Rotational kinetic energy matrix elements

The rotational energy operator $\hat{T}_{\mathrm{rot}}$ can be written as

$$\hat{T}_{\mathrm{rot}}=\frac{1}{2}\hat{\boldsymbol{P}}_{\mathrm{total}}^{\mathrm{T}}\hat{\boldsymbol{O}}_{\mathrm{rot}}(\boldsymbol{Q})\hat{\boldsymbol{P}}_{\mathrm{total}}.\tag{S6}$$

The explicit form of the rotational projection operator $\hat{\boldsymbol{O}}_{\mathrm{rot}}(\boldsymbol{Q})$ is given by Miller *et al.* (see Eq. (4.10a) in ref. S1) as

$$\hat{\boldsymbol{O}}_{\mathrm{rot}}(\boldsymbol{Q})=\tilde{\boldsymbol{Q}}^{\mathrm{T}}\begin{pmatrix}\boldsymbol{I}(\boldsymbol{Q})^{-1} & \boldsymbol{I}(\boldsymbol{Q})^{-1} & \cdots\\ \boldsymbol{I}(\boldsymbol{Q})^{-1} & \boldsymbol{I}(\boldsymbol{Q})^{-1} & \cdots\\ \vdots & \vdots & \ddots\end{pmatrix}\tilde{\boldsymbol{Q}},\tag{S7}$$

$$\tilde{\boldsymbol{Q}} = \begin{pmatrix} 0 & -\sqrt{m^{(1)}}\hat{z}^{(1)} & \sqrt{m^{(1)}}\hat{y}^{(1)} & & & & \\ \sqrt{m^{(1)}}\hat{z}^{(1)} & 0 & -\sqrt{m^{(1)}}\hat{x}^{(1)} & & \boldsymbol{0} & & \cdots \\ -\sqrt{m^{(1)}}\hat{y}^{(1)} & \sqrt{m^{(1)}}\hat{x}^{(1)} & 0 & & & & \\ & & & 0 & -\sqrt{m^{(2)}}\hat{z}^{(2)} & \sqrt{m^{(2)}}\hat{y}^{(2)} & \\ & \boldsymbol{0} & & \sqrt{m^{(2)}}\hat{z}^{(2)} & 0 & -\sqrt{m^{(2)}}\hat{x}^{(2)} & \cdots \\ & & & -\sqrt{m^{(2)}}\hat{y}^{(2)} & \sqrt{m^{(2)}}\hat{x}^{(2)} & 0 & \\ & \vdots & & & \vdots & & \ddots \end{pmatrix}, \quad \text{(S8)}$$

where $\boldsymbol{I}$ is the moment of inertia. Here, we denote the Cartesian coordinates j=x,y,z of the ath atom as $j^{(a)}$, and the mass of the ath atom as $m^{(a)}$. Since it is difficult to calculate the matrix elements of $\hat{T}_{\mathrm{rot}}$ exactly using Eq. (S6), we introduce the following approximation:

$$\langle G_i|\hat{T}_{\mathrm{rot}}|G_{i'}\rangle = \left\langle G_i\middle|\frac{1}{2}\hat{\boldsymbol{P}}_{\mathrm{total}}^{\mathrm{T}}\hat{\boldsymbol{O}}_{\mathrm{rot}}(\boldsymbol{Q})\hat{\boldsymbol{P}}_{\mathrm{total}}\middle|G_{i'}\right\rangle \approx \left\langle G_i\middle|\frac{1}{2}\hat{\boldsymbol{P}}_{\mathrm{total}}^{\mathrm{T}}\hat{\boldsymbol{O}}_{\mathrm{rot}}(\boldsymbol{Q}_{i,i'})\hat{\boldsymbol{P}}_{\mathrm{total}}\middle|G_{i'}\right\rangle. \quad \text{(S9)}$$

The matrix elements of $\hat{T}_{\mathrm{rot}}$ is then evaluated as

$$\langle G_i|\hat{T}_{\mathrm{rot}}|G_{i'}\rangle \approx \hbar^2\{-2\boldsymbol{K}_{i,i'}^{\mathrm{T}}\boldsymbol{O}_{\mathrm{rot}}(\boldsymbol{Q}_{i,i'})\boldsymbol{K}_{i,i'} + \mathrm{Tr}[\boldsymbol{O}_{\mathrm{rot}}(\boldsymbol{Q}_{i,i'})\boldsymbol{\alpha}_i(\boldsymbol{\alpha}_i+\boldsymbol{\alpha}_{i'})^{-1}\boldsymbol{\alpha}_{i'}]\}\langle G_i|G_{i'}\rangle, \quad \text{(S10)}$$

where $\boldsymbol{K}_{i,i'}$ is a 3N-dimensional vector defined by

$$\boldsymbol{K}_{i,i'} = \boldsymbol{\alpha}_i(\boldsymbol{\alpha}_i+\boldsymbol{\alpha}_{i'})^{-1}\boldsymbol{\alpha}_{i'}(\boldsymbol{Q}_i-\boldsymbol{Q}_{i'}). \quad \text{(S11)}$$

S3. Comparison with the previous version of the SBG expansion

Before introducing machine learning techniques (PCA and GPR), we propose two improvements to the SBG expansion method to enhance its computational accuracy:

(i) Vibrational kinetic energy operator $\hat{T}_{\mathrm{vib}}$

In our previous study,[S2] the rotational contributions contaminating the vibrational kinetic energy operator were partly removed by aligning molecules so as to avoid angular momentum generation during structural shifts. In the present study, in addition to this alignment, we further improve the process of removing the rotational contributions by defining the rotational energy operator $\hat{T}_{\mathrm{rot}}$ as in Eq. (S6). The vibrational kinetic energy operator $\hat{T}_{\mathrm{vib}}$ is then defined by subtracting the translational and rotational energy operators, $\hat{T}_{\mathrm{tra}}$ and $\hat{T}_{\mathrm{rot}}$, from the total kinetic energy operator $\hat{T}_{\mathrm{tot}}$ as shown in Eq. (7) in the main text.

(ii) Width parameters $\boldsymbol{\alpha}_i$

In our previous study,[S2] we defined $\boldsymbol{\alpha}_i$ as a block diagonal matrix with N diagonal blocks determined by 3×3 atomic Hessian matrices, which omit interatomic coupling motions. Then, $\boldsymbol{\alpha}_i$ were calculated using the Hessian matrices only at equilibrium structures. For the bases placed along or around the IRC, $\boldsymbol{\alpha}_i$ were set as a weighted average of those at the equilibrium structures.

In this study, to incorporate the interatomic coupling effects, $\boldsymbol{\alpha}_i$ are defined using the full $3N\times3N$ Hessian matrix as in Eq. (3). Moreover, to adequately capture the changes in the curvature of PESs, we calculate $\boldsymbol{\alpha}_i$ for each basis individually using the corresponding local Hessian matrix.

To verify the effects of these improvements, we calculated the vibrational eigenenergies of H_3O^+. We placed five bases on the IRC of the umbrella inversion and added first- and second-order shifted bases along all local normal modes.

The results before and after incorporating the improvements are compared in **Table S3**. The vibrational energies obtained using the previous version of the SBG expansion qualitatively reproduce the experimental results[S3–S5] but exhibit some error. Incorporating the two improvements progressively decreases the error, yielding highly accurate results especially when both improvements (i) and (ii) are introduced. In the main text, the updated version of the SBG expansion incorporating both improvements (i) and (ii) is adopted.

Table S3. Vibrational energies and their root mean square error (RMSE) from the experimental values[S3–S5] in units of cm^{-1} calculated using the SBG expansion method proposed in our previous study and its two updated versions introduced in this Letter: one incorporating improvement (i), and the other incorporating improvements (i) and (ii). The latter is used in the main text.

	Previous version[S2]	Improvement (i)	Improvements (i) and (ii)
0^-	58.47	56.87	47.52
ν_2^+	603.93	606.20	593.83
ν_2^-	1045.20	1050.23	989.20
$2\nu_2^+$	1666.99	1587.24	1492.20
RMSE	106.43	74.54	20.62

S4. Orthogonalization of principal components to the IRC

In a similar manner to the local normal mode approach, principal components {PCn} can be orthogonalized to the IRC at the center of each basis on the IRC. We remove the component parallel to the IRC from the covariance matrix $\boldsymbol{C}$ using the projection matrix onto the direction of the IRC, $\boldsymbol{O}_{\mathrm{IRC}}(\boldsymbol{Q}_i)$:

$$\boldsymbol{C}_{\mathrm{orth}}(\boldsymbol{Q}_i) = [\mathbf{1} - \boldsymbol{O}_{\mathrm{IRC}}(\boldsymbol{Q}_i)]^{\mathrm{T}}\boldsymbol{C}[\mathbf{1} - \boldsymbol{O}_{\mathrm{IRC}}(\boldsymbol{Q}_i)]. \quad \text{(S12)}$$

By diagonalizing $\boldsymbol{C}_{\mathrm{orth}}(\boldsymbol{Q}_i)$, we can obtain the eigenvectors PC$n_{\mathrm{orth}}(\boldsymbol{Q}_i)$ orthogonal to the IRC (with the eigenvalues $\lambda_{n,\mathrm{orth}}(\boldsymbol{Q}_i) > 0$).

The calculated $\lambda_{n,\mathrm{orth}}(\boldsymbol{Q}_{\mathrm{eq}})$ and the characters of PC$n_{\mathrm{orth}}(\boldsymbol{Q}_{\mathrm{eq}})$ at the equilibrium structure of H_3O^+ are shown in **Table S4**. Before orthogonalizing PCn to the IRC, two coordinates have significant λ_n values (see **Table 2** in the main text); after the transformation by Eq. (S12), only one coordinate has a significant $\lambda_{n,\mathrm{orth}}(\boldsymbol{Q}_{\mathrm{eq}})$ value. The $\lambda_{n,\mathrm{orth}}(\boldsymbol{Q}_{\mathrm{eq}})$ corresponding to PC1 in **Table 2**, i.e., the umbrella inversion, is approximately zero since PC1 is almost parallel to the IRC. As a result, the OH symmetric stretching, which is PC2 in **Table 2**, becomes PC$1_{\mathrm{orth}}(\boldsymbol{Q}_{\mathrm{eq}})$ with the largest $\lambda_{n,\mathrm{orth}}(\boldsymbol{Q}_{\mathrm{eq}})$ value. Umbrella inversion is mixed with other vibrations as well as rotations and translations to form PC$2_{\mathrm{orth}}(\boldsymbol{Q}_{\mathrm{eq}})$–$12_{\mathrm{orth}}(\boldsymbol{Q}_{\mathrm{eq}})$.

Table S4. Principal components $\mathrm{PC}n_{\mathrm{orth}}(\boldsymbol{Q}_{\mathrm{eq}})$ orthogonalized to the IRC at the equilibrium structure and the corresponding eigenvalues $\lambda_{n,\mathrm{orth}}(\boldsymbol{Q}_{\mathrm{eq}})$.

	$\lambda_{n,\mathrm{orth}}(\boldsymbol{Q}_{\mathrm{eq}})/\lambda_{1,\mathrm{orth}}(\boldsymbol{Q}_{\mathrm{eq}})$	Character
$\mathrm{PC1}_{\mathrm{orth}}(\boldsymbol{Q}_{\mathrm{eq}})$	1	OH symmetric stretching
$\mathrm{PC2}_{\mathrm{orth}}(\boldsymbol{Q}_{\mathrm{eq}})$–$12_{\mathrm{orth}}(\boldsymbol{Q}_{\mathrm{eq}})$	$<10^{-5}$	OH asymmetric stretching,<br>HOH bending,<br>Umbrella inversion,<br>Rotation, Translation

The vibrational energies computed for the case where bases are added along $\mathrm{PC1}_{\mathrm{orth}}(\boldsymbol{Q}_i)$ are shown in the second column of **Table S5**. Other computational details are the same as for the results presented in the main text. Since $\mathrm{PC1}_{\mathrm{orth}}(\boldsymbol{Q}_i)$ is nearly identical to PC2 in **Table 2**, the bases are added at similar positions to those along PC2 as illustrated in **Fig. S1**. Consequently, the calculated vibrational energies are close to the values obtained with additional bases along PC2 (the fifth column of **Table S5**). To improve the computational accuracy, adding bases in the direction parallel to the IRC (denoted as $\mathrm{v}_{\mathrm{IRC}}(\boldsymbol{Q}_i)$), which approximately corresponds to PC1 in **Table 2**, is expected to be more effective than adding bases along $\mathrm{PC2}_{\mathrm{orth}}(\boldsymbol{Q}_i)$. The results in the two cases are compared in the third and fourth columns of **Table S5**. As expected, the accuracy improves only little with additional bases along $\mathrm{PC1}_{\mathrm{orth}}(\boldsymbol{Q}_i)$ and $\mathrm{PC2}_{\mathrm{orth}}(\boldsymbol{Q}_i)$. On the other hand, much fewer additional bases along $\mathrm{v}_{\mathrm{IRC}}(\boldsymbol{Q}_i)$ and $\mathrm{PC1}_{\mathrm{orth}}(\boldsymbol{Q}_i)$ significantly reduces the error from the experimental data. The results in the latter case are almost identical to those obtained by adding bases along PC1 and PC2 (the fourth column of **Table 1** in the main text). These confirm that

computational accuracy is unchanged as long as the basis arrangement is similar with or without the orthogonalization of principal components to the IRC.

Table S5. Vibrational energies in units of cm^{-1} calculated with four types of basis arrangements. Additional bases are placed along one or two of the following coordinates: two principal components orthogonal to the IRC ($PC1_{orth}(\boldsymbol{Q}_i)$ and $PC2_{orth}(\boldsymbol{Q}_i)$), the second principal component in **Table 2** (PC2), and the direction parallel to the IRC ($\nu_{IRC}(\boldsymbol{Q}_i)$).

	$PC1_{orth}$	ν_{IRC} + $PC1_{orth}$	$PC1_{orth}$, 2_{orth}	PC2
No. of bases	25	33	65	25
ZPE	7478.72	7476.38	7472.85	7478.93
0^{-}	46.56	45.43	47.94	47.37
ν_2^{+}	614.64	596.53	613.45	619.80
ν_2^{-}	1137.72	991.74	1123.97	1169.25
$2\nu_2^{+}$	1926.11	1492.09	1904.45	1986.57
RMSE	243.69	22.32	231.06	277.74

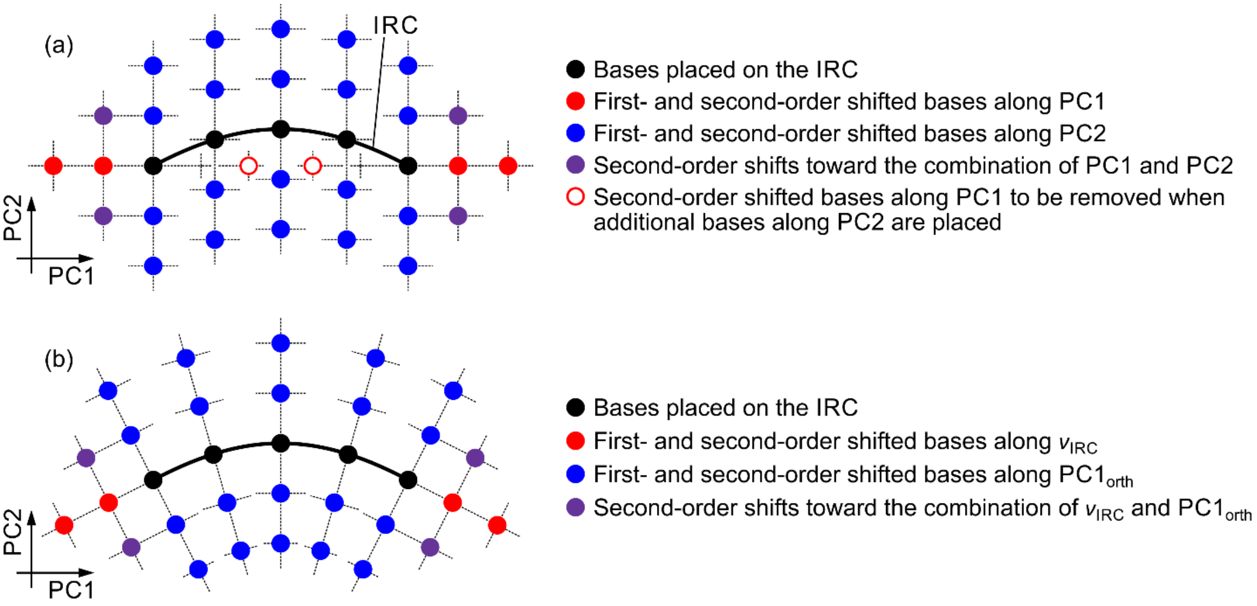

Figure S1. Schematic illustrations of the arrangement of additional bases (red, blue and purple circles) along (a) PC1 and PC2, and (b) $\mathrm{v_{IRC}}(\boldsymbol{Q}_i)$ and $\mathrm{PC1_{orth}}(\boldsymbol{Q}_i)$, added around the bases on the IRC (black circles). Dashed lines indicate the directions of PC1 and PC2 in panel (a) and those of $\mathrm{v_{IRC}}(\boldsymbol{Q}_i)$ (parallel to the IRC) and $\mathrm{PC1_{orth}}(\boldsymbol{Q}_i)$ in panel (b). Since most of the bases to be added along PC1 and $\mathrm{v_{IRC}}(\boldsymbol{Q}_i)$ have large overlaps with those on the IRC or other additional ones, only a small number of bases are ultimately added along these directions.

S5. Potential interpolation by GPR with a large number of bases

Potential interpolation is applicable to any basis set for the SBG expansion. We apply GPR to the basis set with additional bases along all local normal modes, which consists of a larger number of bases than the one used for validating the GPR interpolation in the main text. The RMSEs in potential energies $V(\boldsymbol{Q}_{i,i'})$ and Hessian terms $\frac{1}{4}\mathrm{Tr}\left[(\boldsymbol{\alpha}_i + \boldsymbol{\alpha}_{i'})^{-1}V''(\boldsymbol{Q}_{i,i'})\right]$, evaluated from the comparison between the estimated values and those obtained by QC calculations, and the RMSE

in vibrational energies from the experimental results[S3–S5] are shown in **Table S6**, along with those for the case where additional bases are placed along PC1 and PC2. The errors in potential energies and Hessian terms increase compared to those with the smaller number of bases. This is because the estimation errors become large at middle points between two distant bases due to the lack of nearby bases, i.e., training data, around the points where the estimation is performed. Since the effect of the estimation errors on the vibrational energy calculation is suppressed by the small overlap integral between the distant bases as shown in Eq. (8), the calculated vibrational energies qualitatively reproduce experimental results. However, the error in the vibrational energies is also larger than in the case with the fewer bases (the second column of **Table S6**). More sophisticated models, such as neural networks, will further improve the estimation accuracy for basis sets with a large number of bases.

Table S6. RMSEs in potential energies, Hessian terms, and vibrational energies in units of cm^{-1}. The first two of them are evaluated from the comparison between the GPR estimated values and those obtained by QC calculations, while the last one is the error from experimental values.[S3–S5]

	PC1, 2	All vib.
No. of bases	33	329
No. of QC calc.	19	195
RMSE in potential energies	12.02	115.28
RMSE in Hessian terms	2.59	13.53
RMSE in vibrational energies	28.79	45.30

S6. Vibrationally excited states for the modes other than the umbrella inversion

An advantage of the SBG expansion is the flexibility of the basis arrangement. It is possible to place SBGs along any dynamical path, which is not necessarily an IRC. Auxiliary coordinates utilized in the basis set construction schemes in the main text are not restricted to principal components but can be arbitrary vibrational coordinates. For instance, local normal coordinates are a reasonable choice as auxiliary coordinates when vibrationally excited states for the modes other than the umbrella inversion are desired. Even in this case, bases are necessary on the IRC because the excited states also exhibit a tunnel splitting. The basis set suitable for this purpose is the "All vib." set in **Table 1** in the main text, consisting of bases along the tunneling IRC and all local normal modes. The calculated vibrational energies are provided in **Table S7**, together with the experimental[S6–S8] and RVIB4[S9] values. The accuracy of our tunnel splitting results is comparable to RVIB4 for all three modes, i.e., ν_1 (OH symmetric stretching), ν_3 (OH asymmetric stretching), and ν_4 (HOH bending). If one focuses on a particular excited state, the accuracy can be further improved by adding bases on paths along the relevant mode. Conversely, redundant bases placed along irrelevant modes can be excluded from the basis set to reduce computational costs. The SBG expansion is thus a general and convenient method for describing arbitrary molecular motion.

Table S7. Calculated energies of vibrationally excited states for the modes other than the umbrella inversion (ν_2) in units of cm^{-1}. The labels ν_1, ν_3, and ν_4 represent the fundamentals of the OH symmetric stretching, OH asymmetric stretching, and HOH bending modes, respectively. The symbols +/− denote the parity of a pair of tunneling states.

	All vib.			Exp.			RVIB4[S9]		
	+	−	split	+	−	split	+	−	split
ν_4	1635.79	1695.84	60.05	1625.95[S6]	1693.87[S6]	67.92	1623	1673	50
	1634.48	1696.48	62.00				1623	1673	50
ν_1	3509.59	3542.27	32.68	3445.00[S7]	3491.17[S7]	46.17	3386	3418	32
ν_3	3532.47	3581.99	49.52	3535.56[S8]	3574.29[S8]	38.73	3522	3550	28
	3533.71	3583.99	50.28				3522	3550	28

REFERENCES

(S1) Miller, W. H.; Handy, N. C.; Adams, J. E. Reaction path Hamiltonian for polyatomic molecules. *J. Chem. Phys.* **1980**, *72* (1), 99−112.

(S2) Suzuki, K.; Kanno, M.; Koseki, S.; Kono, H. A Structure-Based Gaussian Expansion for Quantum Reaction Dynamics in Molecules: Application to Hydrogen Tunneling in Malonaldehyde. *J. Phys. Chem. A* **2023**, *127* (18), 4152−4165.

(S3) Liu, D.-J.; Haese, N. N.; Oka, T. Infrared spectrum of the ν_2 vibration-inversion band of H_3O^+. *J. Chem. Phys.* **1985**, *82* (12), 5368−5372.

(S4) Liu, D.-J.; Oka, T. Experimental Determination of the Ground-State Inversion Splitting in H_3O^+. *Phys. Rev. Lett.* **1985**, *54* (16), 1787−1789.

(S5) Davies, P. B.; Johnson, S. A.; Hamilton, P. A.; Sears, T. J. Infrared diode laser spectroscopy of the $\nu_2(2^+ \leftarrow 1^-)$ band of H_3O^+. *Chem. Phys.* **1986**, *108* (3), 335−341.

(S6) Gruebele, M; Polak, M.; Saykally, R. J. A study of the structure and dynamics of the hydronium ion by high resolution infrared laser spectroscopy. II. The ν_4 perpendicular bending mode of $H_3{}^{16}O^+$. *J. Chem. Phys.* **1987**, *87* (6), 3347–3351.

(S7) Tang, J.; Oka, T. Infrared Spectroscopy of H_3O^+: The ν_1 Fundamental Band. *J. Mol. Spectrosc.* **1999**, *196* (1), 120-130.

(S8) Begemann, M. H.; Saykally, R. J. A study of the structure and dynamics of the hydronium ion by high resolution infrared laser spectroscopy. I. The ν_3 band of $H_3{}^{16}O^+$. *J. Chem. Phys.* **1985**, *82* (8), 3570–3579.

(S9) Huang, X.; Carter, S.; Bowman, J. M. Ab Initio Potential Energy Surface and Vibrational Energies of H_3O^+ and Its Isotopomers. *J. Phys. Chem. B* **2002**, *106* (33), 8182−8188.
