# Supplementary material for: Machine Learning-Enhanced Structure-Based Gaussian Expansion for Efficient Wavepacket Calculations
Source: J Phys Chem Lett. 2025 Jun 7;16(24):5986–92. doi: 10.1021/acs.jpclett.5c01254 (PMC12183755; doi:10.1021/acs.jpclett.5c01254)
Supplement: Supplementary file 2 [file jz5c01254_si_002.pdf]

jz-2025-01254j.R1

Name: Peer Review Information for "Machine Learning-Enhanced Structure-Based Gaussian Expansion for Efficient Wavepacket Calculations"

First Round of Reviewer Comments

Reviewer: 1

Comments to the Author

This manuscript by Koshiba et al. reports on a modification of a method they have previously published in JPC A. The methodology looks fine, though the test case they use isn't particularly demanding. While it's a reasonably solid paper, I am not certain that it meets the breadth of interest or urgency expected from a JPCL paper.

While it is seemingly impressive that reasonable results can be obtained from only 19 ab initio calculations (noting that these are full Hessian calculations), the test system used is quite simple. It's essentially 2D dynamics, which is borne out by the PCA undertaken by the authors. The authors only present v2 results, which seems quite restricted. They compare their v2 results with a full dimensional model result. But what about other modes, and other methods? I would not be surprised to discover a low dimensionality calculation (reaction path Hamiltonian-like) or e.g. MCTDH on a Shepard interpolation potential from a similar number of points to be similarly accurate for v2.

Equation (3) is difficult to interpret. V''(Qi) is a matrix. There is no such operation as the square root of an indefinite matrix. What factorisation are the authors referring to here?

The PCA result for a symmetric inversion is not surprising. More interesting would have been an asymmetric reaction that does not reduce to two effective degrees of freedom.

It is not surprising that the αs cannot be allowed to become too small. No system likes to be described with huge width Gaussians in the LHA. I suspect the authors will have more difficulties with less harmonic potentials.

Reviewer: 2

Comments to the Author

The author presents an extension of their previous work used structured based basis Gaussian (SBG)functions to describe vibrational dynamics for an isomerization process. Instead of placing the SBG's along the IRC pathway connecting the two wells in an intuitive way, they propose here to use PCA to take into account the main directions of motion along the IRC. They also propose to use machine learning (Gaussian Process Regression) to interpolate between computed points of the potential. The approach is illustrated on the umbrella inversion of H3O+. The paper describes in details all the technical points and the respective advantages of PCA for selecting geometries where to place Gaussian in addition to sampling the IRC and the GPR for interpolation of the potential at middle points between the center of the gaussian basis functions. The results are benchmarked by computing the vibrational frequencies of specific modes involved in the inversion obtained by diagonalizing the vibrational Hamiltonian built from the SBG functions. The approach is interesting to a wide range of molecular dynamicists and appears to be accurate in the case of the umbrella inversion of H3O+. To appreciate its potential, it would be good, already in the present paper, to offer an estimation of the scaling, not so much with the number of atoms as is done in the concluding paragraph, than with the number of modes needed in addition to the IRC and the type of reaction described by the IRC. The eigenvalues of the PCA analysis reported in Table 2 appear to decrease very fast. Is this a general result intrinsic to the determination of the IRC or is it specific to the kind of reaction coordinate for the umbrella motion. It would be valuable for the paper that the authors discuss this point.

Author's Response to Peer Review Comments:

Dear Editor,

I am sending herewith a revised manuscript of jz-2025-01254j entitled “Machine Learning-Enhanced Structure-Based Gaussian Expansion for Efficient Wavepacket Calculations” by Takumi Koshiba, Manabu Kanno, Fuminori Misaizu, and Hirohiko Kono, which we submitted for publication in the Journal of Physical Chemistry Letters.

We found the comments raised by Reviewers 1 and 2 very helpful and revised our manuscript accordingly. In the following, our replies to the comments and the changes made in the manuscript are written in blue and red, respectively. In a marked copy of the manuscript, our revisions are also indicated in red.

Reviewer 1:

Comment:
This manuscript by Koshiba et al. reports on a modification of a method they have previously published in JPC A. The methodology looks fine, though the test case they use isn’t particularly demanding. While it’s a reasonably solid paper, I am not certain that it meets the breadth of interest or urgency expected from a JPCL paper.

While it is seemingly impressive that reasonable results can be obtained from only 19 ab initio calculations (noting that these are full Hessian calculations), the test system used is quite simple. It’s essentially 2D dynamics, which is borne out by the PCA undertaken by the authors.

Author reply:
We would like to thank the reviewer for recognizing the usefulness of our methodology. As he/she pointed out, the inversion in H_3O^+ is essentially 2D dynamics represented by the umbrella and OH symmetric stretching vibrations. This has been shown, e.g., by an elaborate 2D model study of Miani *et al*. [Miani, A.; Beddoni, A.; Pesonen, J.; Halonen, L. CCSD(T) inversion spectrum for H_3O^+. *Chem. Phys. Lett.* **2002**, *363* (1−2), 52−56], and our PCA succeeded in automatically identifying the two dominant degrees of freedom. We cited the above-mentioned paper by Miani *et al*. as Ref. 24 and added the following sentence on pp. 13–14 in the revised manuscript:

“This is consistent with a two-dimensional model developed by Miani *et al*., which suggested that the umbrella and OH symmetric stretching vibrations are sufficient to accurately describe the inversion motion.[24]”

In response to the reviewer’s point that the test system used is too simple, we have also applied our SBG approach to hydrogen tunneling in 9-hydroxyphenalenone (9HP-d_0) and its asymmetrically deuterated species (9HP-d_1), which are much larger than H_3O^+. Even for such a large system consisting of 23 atoms, the calculated results successfully converged as the number of principal components (PCn) was increased in order of decreasing eigenvalues (variances) λ_n. The first four principal components (PC1 to PC4) were required to achieve convergence, indicating that this system involves dynamics more complex than the previously studied 2D cases. Convergent results were obtained using only 289 SBG bases, suggesting a quite low computational cost. To explain these results, we added two paragraphs on pp. 17–18. For details, please read the paragraphs. They begin as follows:

“Finally, to test the feasibility of the present method for systems with far more than 10 atoms, …”

Besides, we added

“We also confirmed the feasibility for larger systems through the applications to intramolecular hydrogen transfer in 9-hydroxyphenalenone and its asymmetrically deuterated species.”

in the Abstract on p. 2 and

“A small number of essential coordinates (including nontrivial ones) can also be identified automatically in large and asymmetric systems like 9HP-d_0 and 9HP-d_1, thus enabling efficient calculations of many-dimensional wavepackets.

in the last (summary) paragraph of the main text on pp. 19–20.

Comment:

The authors only present ν2 results, which seems quite restricted. They compare their ν2 results with a full dimensional model result. But what about other modes, and other methods? I would

not be surprised to discover a low dimensionality calculation (reaction path Hamiltonian-like) or e.g. MCTDH on a Shepard interpolation potential from a similar number of points to be similarly accurate for ν2.

Author reply:

Following the suggestion, we presented the energies of vibrationally excited states for the other modes of H_3O^+, which had already been obtained using the "All vib." basis set consisting of bases along the IRC and all local normal modes, in Table S7 of the SI. The accuracy of our results is comparable to RVIB4 for all three modes, i.e., ν_1 (OH symmetric stretching), ν_3 (OH asymmetric stretching), and ν_4 (HOH bending). This exemplifies that our SBG approach is not limited to specific reaction coordinates but is a general and convenient method for describing arbitrary molecular motion. We added the following paragraph on p. 17:

"So far, we have focused on the umbrella inversion and therefore derived principal components from the IRC to prepare the optimal basis set for this mode. A great advantage of the SBG expansion is that one can adopt arbitrary appropriate paths and auxiliary coordinates for basis set construction, depending on the problem of interest. For instance, vibrationally excited states and tunnel splittings of the other modes in H_3O^+ can be adequately described by distributing bases in the corresponding directions (see the results for the "All vib." case in Section S6 of the SI)."

For details of these results, please see Section S6, newly added in the SI.

The reviewer also presumed the existence of other methods, especially low-dimensional ones, with a similar accuracy for ν_2 (umbrella inversion) from a similar number of grid points. Indeed, the above-mentioned 2D model by Miani *et al.* is similarly accurate for ν_2 at the same CCSD(T)/aug-cc-pVTZ level. However, they used as many as 100 grid points for the 2D results. We would like to emphasize that our machine learning-enhanced SBG expansion required only 19 ab initio calculations to obtain full-dimensional nuclear wavefunctions of H_3O^+. Moreover, as noted above, we needed no more than 289 SBGs to simulate hydrogen tunneling in 9HP consisting of 23 atoms. The computational efficiency of our method far exceeds that of other methods, which is particularly pronounced for large systems. To make this point clearer, we added the following sentences (for H_3O^+) on p. 16:

“Compared to the study by Miani *et al.*, which required around 100 QC calculations for a limited two-dimensional model,[24] our approach achieves comparable results with substantially fewer calculations, underscoring its computational efficiency.”

We also added the following sentences (for 9HP) on p. 18:

“The total number of bases in the “PC1–4” basis set is 289. It is much smaller than 9×10^4 for the case with additional first- and second-order shifted bases along all 63 vibrational degrees of freedom.”

Comment:

Equation (3) is difficult to interpret. V"(Qi) is a matrix. There is no such operation as the square root of an indefinite matrix. What factorisation are the authors referring to here?

Author reply:

By replacing near-zero eigenvalues with a positive constant V_{const}'' and flipping the signs of negative eigenvalues, $\boldsymbol{V}''(\boldsymbol{Q}_i)$ becomes a positive-definite matrix. According to linear algebra, a positive-definite matrix has one unique positive-definite square root (principal square root). The principal square root of $\boldsymbol{V}''(\boldsymbol{Q}_i)$ is given by $\sqrt{\boldsymbol{V}''(\boldsymbol{Q}_i)} = \boldsymbol{U}(\boldsymbol{Q}_i)\sqrt{\boldsymbol{\Lambda}(\boldsymbol{Q}_i)}\boldsymbol{U}(\boldsymbol{Q}_i)^{\mathrm{T}}$, where $\boldsymbol{U}(\boldsymbol{Q}_i)$ is the orthogonal matrix composed of the eigenvectors of $\boldsymbol{V}''(\boldsymbol{Q}_i)$ and $\sqrt{\boldsymbol{\Lambda}(\boldsymbol{Q}_i)}$ is the diagonal matrix with diagonal elements equal to the positive square roots of the eigenvalues of $\boldsymbol{V}''(\boldsymbol{Q}_i)$. On p. 5, we modified the second to fourth sentences below Eq. (3) as follows:

“$\boldsymbol{V}''(\boldsymbol{Q}_i)$ has six zero-eigenvalues corresponding to the translation and rotation, making Gaussian integrals incomputable. Moreover, at non-equilibrium structures, it may include negative eigenvalues corresponding to imaginary-frequency vibrational modes, which preclude the definition of $\sqrt{\boldsymbol{V}''(\boldsymbol{Q}_i)}$. To avoid these problems, we convert $\boldsymbol{V}''(\boldsymbol{Q}_i)$ into a positive-definite matrix by the following operations: the eigenvalues close to zero are replaced with a finite positive

constant V_{const}'', and the signs of the negative eigenvalues are flipped to accurately capture the steep shape of $\chi(\boldsymbol{Q}, t)$ in the potential region with a negative curvature."

Comment:

The PCA result for a symmetric inversion is not surprising. More interesting would have been an asymmetric reaction that does not reduce to two effective degrees of freedom.

Author reply:

Following the suggestion, we calculated a tunnel splitting for hydrogen transfer in asymmetrically deuterated 9HP (9HP-d_1) for comparison with that in the symmetric unsubstituted one (9HP-d_0). As noted in our reply to the first comment, hydrogen transfer in 9HP can be viewed as 4D dynamics, and we successfully obtained convergent results at a low computational cost. Please see pp. 17–18.

In recent years, there has been a growing need for theoretical approaches capable of investigating chemical reactions in large, complex molecular systems, with explicit inclusion of quantum effects. One notable example is the study of deuterated pharmaceuticals, where secondary isotope effects — arising from deuterium substitution at positions not directly involved in bond-making or -breaking processes — remain poorly understood. Whether or not these effects manifest depends strongly on the molecular framework and the site of substitution. The machine learning-enhanced SBG method proposed in this Letter has the potential to serve as a powerful tool for exploring such effects and elucidating their underlying mechanisms. Please see p. 18 for discussions on the magnitude of secondary isotope effects on the ground-state tunnel splitting in 9HP.

Comment:

It is not surprising that the αs cannot be allowed to become too small. No system likes to be described with huge width Gaussians in the LHA. I suspect the authors will have more difficulties with less harmonic potentials.

Author reply:

We would like to thank the reviewer for his/her comment that got to the point. An idea to ensure the local harmonic approximation (LHA) for such potentials is adjusting the width parameter $\boldsymbol{\alpha}_i$ in Eq. (3), e.g., by multiplying a scaling factor, which will result in narrow Gaussians. Indeed, we

demonstrated in our previous studies (Refs. 10 and 11) that the LHA holds accurately with narrow Gaussians for a rounded rectangular potential barrier (1D) and multiple wells consisting of local quartic potentials (1D to 3D). The price of using narrow widths is an increase in the number of Gaussians. However, as demonstrated in the manuscript, the number of Gaussians and that of ab initio calculations can be dramatically reduced by introducing PCA and GPR. This suppresses an increase in computational cost caused by using narrow Gaussians and thus enables an application of our method to less harmonic systems. We added the following sentences in the last (summary) paragraph on p. 20:

"The machine learning-enhanced SBG expansion is expected to be useful for challenging systems such as those with strongly anharmonic PESs; PCA and GPR suppress an increase in computational cost due to the use of multiple narrow Gaussians necessitated by the anharmonicity."

Reviewer 2:

Comment:

The author presents an extension of their previous work used structured based basis Gaussian (SBG) functions to describe vibrational dynamics for an isomerization process. Instead of placing the SBG's along the IRC pathway connecting the two wells in an intuitive way, they propose here to use PCA to take into account the main directions of motion along the IRC. They also propose to use machine learning (Gaussian Process Regression) to interpolate between computed points of the potential. The approach is illustrated on the umbrella inversion of H_3O^+. The paper describes in details all the technical points and the respective advantages of PCA for selecting geometries where to place Gaussian in addition to sampling the IRC and the GPR for interpolation of the potential at middle points between the center of the gaussian basis functions. The results are benchmarked by computing the vibrational frequencies of specific modes involved in the inversion obtained by diagonalizing the vibrational Hamiltonian built from the SBG functions. The approach is interesting to a wide range of molecular dynamicists and appears to be accurate in the case of the umbrella inversion of H_3O^+. To appreciate its potential, it would be good, already in the present paper, to offer an estimation of the scaling, not so much with the number of atoms as is done in the concluding paragraph, than with the number of modes needed in addition to the IRC and the type of reaction described by the IRC. The eigenvalues of the PCA analysis reported in Table 2 appear to decrease very fast. Is this a general result intrinsic to the determination of the IRC or is it specific to the kind of reaction coordinate for the umbrella motion. It would be valuable for the paper that the authors discuss this point.

Author reply:

We appreciate the high recommendation "This paper is publishable subject to minor revisions noted. Further review is not needed." by the reviewer. The rapid decrease of the eigenvalues λ_n is not unique to the umbrella motion but is commonly observed in various systems we have studied. Please see Fig. 2(b), newly added on p. 19, for the examples of hydrogen transfer in 9HP. This is one of the reasons why we believe PCA facilitates the application of the SBG expansion to large molecules. We added the following sentences on p. 18:

"As in the case of H_3O^+, λ_n exhibits an exponential decay with increasing n. We have studied some other systems, and the rapid decay is a common trend. This suggests that a limited number of PCn are sufficient even for such large molecules."

Finally, we would like to express our sincere gratitude again to the two reviewers for their valuable comments, which significantly improved the quality of this Letter. We hope that the revised manuscript is now acceptable for publication in the Journal of Physical

Chemistry Letters.

With best regards,

Manabu Kanno

Corresponding Author:

Dr. Manabu Kanno

Department of Chemistry

Graduate School of Science, Tohoku University

Sendai 980-8578, Japan

Tel: (+81)22-795-7729

E-mail: manabu.kanno.d2@tohoku.ac.jp
